# Zoonotic Ascariasis, United Kingdom

**DOI:** 10.3201/eid1710.101826

**Published:** 2011-10

**Authors:** Richard P. Bendall, Maggie Barlow, Martha Betson, J. Russell Stothard, Peter Nejsum

**Affiliations:** Royal Cornwall Hospital, Truro, UK (R.P. Bendall);; Health Protection Agency, St. Austell, UK (M. Barlow);; Natural History Museum, London, UK (M. Betson, J.R. Stothard);; Liverpool School of Tropical Medicine, Liverpool, UK (M. Betson, J.R. Stothard);; University of Copenhagen, Copenhagen, Denmark (P. Nejsum)

**Keywords:** Ascaris, ascariasis, zoonoses, Cornwall, epidemiology, Enterobius, parasites, United Kingdom, letter

**To the Editor:** *Ascaris lumbricoides/suum* is a complex of closely related enteric roundworms that mainly infect humans and pigs (*1*). Transmission occurs through ingestion of fecally excreted ova. *A. lumbricoides* worms usually infect humans, mainly in regions with poor sanitation, where the environment is contaminated with human feces. In industrialized countries, human ascariasis is uncommon and cases are generally believed to have been imported (*2*). By contrast, *A. suum* infection of pigs occurs worldwide; in the United Kingdom, 3.4%–6.5% of pigs at slaughter have evidence of infection (*3*). Sporadic zoonotic infection with *A. suum* in the industrialized world is described (*4*–*6*) but poorly quantified. We describe probable zoonotic transmission of *Ascaris* spp. roundworms in Cornwall, a rural county in southwestern England.

Incidence rates for ascariasis in Cornwall and the rest of England were calculated from local and national laboratory data. From 2004 through 2008, a total of 18 cases were identified in Cornwall, and 314 from the rest of England were reported to the Health Protection Agency; annual rates were 0.87 and 0.12 cases per 100,000 population, respectively.

From 1995 through 2010, a total of 63 ascariasis cases were identified in Cornwall, and details of patient age, sex, and place of residence were collected. Patients from Cornwall were younger (mean age 22 years) than those from other parts of England (mean age 31 years), and the proportion of patients <5 years of age in Cornwall (35.5%) was greater than that in the rest of England (19.7%). Similar proportions (61% vs. 65%) of patients from Cornwall and England were female.

The possibility of zoonotic transmission in Cornwall was investigated by comparing risk factors for ascariasis and enterobiasis (caused by an enteric helminth that infects only humans). From 1995 through 2010, the laboratory in Cornwall identified 38 cases of *Enterobius* infection. Patient mean age was 24 years (range 1–95 years); 2 (5.7%) patients were <5 years of age and 23 (60.5%) were female. The following risk factors were considered for statistical analysis: age <5 years, female sex, and residence near pig herds. Residence was determined by comparing the postcodes of case-patients with postcodes of pig holdings registered with the Department of Environment, Food and Rural Affairs. The UK postal service allots a maximum of 80 households to a postcode. In rural areas like Cornwall, the number is much smaller. Consequently, sharing a postcode with a pig holding implies proximity to pig herds. Of the 50 ascariasis patients with a Cornwall postcode, 11 (22%) shared that postcode with a pig holding. Of the 35 enterobiasis patients in Cornwall, only 2 (5.7%) shared a postcode with a pig holding.

Odds ratios were calculated for all 3 risk factors, and the Fisher exact test was used to determine their significance. We calculated p values by using 2-tailed models for age and sex and a 1-tailed model to test the association with residence near a pig holding (Table). Significant associations were found for age <5 years (odds ratio 6.42, p = 0.0037) and living near pigs (odds ratio 4.65, p = 0.036) but not for female sex.

**Table:** Risk factors for ascariasis versus enterobiasis in residents of Cornwall, United Kingdom, 1995–2010

Risk factor	Ascariasis, no. with risk factor/total no. cases*	Enterobiasis, no. with risk factor/total no. cases†	Odds ratio (95% confidence interval)	p value‡
Age <5 y	22/62	3/38	6.42 (1.77–23.30)	**0.0037**
Female sex	35/62	22/38	0.845 (0.372–1.920)	0.83
Pig holding within same postcode	11/50	2/35	4.65 (0.962–22.500)	**0.036**

*n = 63 patients. Postcode not available for 12 patients; 1 postcode was outside of Cornwall. Thus, only 50/63 patients were included in postcode analysis. Sex not indicated for 1 patient; age not indicated for 1 patient. Thus, only 62/63 patients were included in age and sex analyses. †n = 38 patients. Postcode outside of Cornwall for 3 patients. Thus, only 35/38 patients were included in postcode analysis. ‡By Fisher exact test. **Boldface** indicates significance.

Further evidence for zoonotic transmission comes from molecular analyses of DNA extracted from 11 *Ascaris* spp. worms recovered from patients in Cornwall. Results were compared by PCR-linked restriction fragment length polymorphism and sequence analysis with those from 35 reference worms from pigs in the United Kingdom, Denmark, Uganda, Guatemala, and the Philippines and from 20 worms from humans in Uganda, Tanzania, and Nepal. We used the PCR-linked restriction fragment length polymorphism method described by Nejsum et al. (*5*). Briefly, the ribosomal internal transcribed spacer region was amplified, and the products were digested with the restriction enzyme *Hae*III and separated into bands by agarose gel electrophoresis. All worms from humans and pigs in the United Kingdom had 3- or 4-banded genotypes, typically found in worms from pigs (*4*,*5*,*7*). By contrast, a 2-banded genotype predominated in worms collected from humans living in *A. lumbricoides*–endemic areas. Similarly, sequence analysis, as described by Nejsum et al. (*8*), of amplified mitochondrial *cox*1 genes using primers by Peng et al. (*9*) showed that all worms from humans in Cornwall clustered with worms from pigs (i.e., had pig-like DNA sequences).

Compared with the rest of the United Kingdom, incidence of human ascariasis is high in Cornwall, especially among children <5 years of age. Because of the retrospective nature of our study, we have little travel or clinical information for these case-patients. However, because such young case-patients would probably not travel much and because postcode data identified place of residence as a risk factor, the data suggest a focus of locally acquired *A. suum* infection in humans in Cornwall. Molecular evidence implicates pigs as the source. Further studies are needed, but if pigs are confirmed to be the source, control and prevention of this emerging infection will probably depend more on modifications of animal husbandry and fecal waste disposal rather than on human sanitation.
